# Influence of Er,Cr:YSGG Laser Irradiation on the Push-Out Bond Strength of Zirconia and Glass Fiber Posts with Radicular Dentin

**DOI:** 10.1155/2019/4869853

**Published:** 2019-10-09

**Authors:** Raneem S. Alofi, Ibraheem F. Alshiddi, Yasser F. AlFawaz, Abdulaziz Alsahhaf, Khulud Abdulrahman Al-Aali, Tariq Abduljabbar, Fahim Vohra

**Affiliations:** ^1^Department of Restorative Dental Sciences, College of Dentistry, King Saud University, Riyadh 11545, Saudi Arabia; ^2^Department of Prosthetic Dental Science, College of Dentistry, King Saud University, Riyadh 11545, Saudi Arabia; ^3^Department of Clinical Dental Sciences, College of Dentistry, Princess Nourah Bint Abdulrahman University, Riyadh, Saudi Arabia; ^4^Research Chair for Biological Research in Dental Health, College of Dentistry, King Saud University, Riyadh 11545, Saudi Arabia

## Abstract

**Objective:**

This *in vitro* study was designed to evaluate the influence of an Er,Cr:YSGG laser on the bond strength of zirconia and glass fiber posts with root dentin.

**Materials and methods:**

Ninety extracted single-rooted human teeth were randomized into 6 groups (*n* = 15/group) on the basis of different posts (zirconia/glass fiber) and Er,Cr:YSGG laser tips (axial and radial). Specimens were prepared for push-out testing with the help of a cutting machine; six slices (2 on each cervical, middle, and apical) of approximately 1 mm thickness were sectioned for all roots on a plane perpendicular to the long axis of the post. All specimens were placed into a universal testing machine with a defined 0.5 mm/min crosshead speed until the maximum failure load was obtained.

**Results:**

The highest mean push-out bond strength of the glass fiber and zirconia groups was achieved with laser treatment. The highest push-out bond strength was achieved with the axial fiber tip (7.63 ± 1.22 MPa), and the lowest was achieved with a radial fiber tip of the glass fiber group (6.98 ± 0.96 MPa). ANOVA showed a statistically significant difference between the groups (*p* = 0.041). The mean push-out bond strength was found to be higher with an axial fiber tip for both cervical and apical segments in the glass fiber and zirconia groups (*p* < 0.05). The independent *t*-test resulted in the overall highest mean push-out bond strength in the apical segments (*p* = 0.026).

**Conclusion:**

Within the limits of the present *in vitro* research study, an enhancement in the push-out bond strength of resin cement, mainly in the cervical region of the root canal, was achieved after irradiation with an Er,Cr:YSGG laser using an axial fiber tip.

## 1. Introduction

Dental posts are used in cases where the tooth structure is extensively deteriorated and requires strong support [1]. The long-term success of endodontic-treated tooth depends on several factors including post surface/interface, root dentin, and the resin cement that helps to tether the post within the root structure [2]. The efficacy of bonding among the three components may aid in the distribution of forces along the root system [3]. Failure of any of these components may lead to improper stress distribution along the tooth structure owing to mastication [4]. Several techniques have been proposed in order to enhance the bond strength of glass fiber post and the dentine surface. As the glass fiber post does not directly bond with the resin surface, microretentive areas are created by roughening to increase the bond strength between the glass fiber and resin. These techniques include chemical roughening including the use of hydrogen peroxide and silane, and mechanical roughening including airborne particle abrasion with aluminum oxide or acid etching [5–7].

The demand for esthetic restorative materials has increased and led to the introduction of yttria-tetragonal zirconia polycrystalline (Y-TZP) ceramics. This dental material is widely used in the fabrication of core materials in fixed bridges, crowns, dental implant abutments, and endodontic posts [8–10]. However, there is a striking difference in the composition and physical properties of silica-based ceramics and zirconia, and acid etching cannot easily create micromechanical tags of resin cements. Therefore, other substitute methods warrant surface alterations in case of zirconia ceramics [11–13]. Several *in vitro* studies have researched the efficacy of different techniques including silica coating and surface modification using lasers [14–16].

Laser technology has been widely used in dental procedures [17–19]. In this regard, different wavelengths have been used to modify the surfaces of restorative materials in order to increase the bond strength. Data from previous studies suggest that laser irradiation enhances surface modification on dental posts, which greatly increases the bond strength with resin cements [20–22]. The laser therapy of a target dental material differs depending on the laser parameters used, such as wavelength. The *in vitro* data seeking to improve the adhesive resin cement-post-dentin interface warrant further investigation. Therefore, this *in vitro* study was designed to evaluate the influence of Er,Cr:YSGG laser on the bond strength of zirconia and glass fiber posts with root dentin.

## 2. Materials and Methods

The experiment was performed with ninety maxillary and mandibular extracted single-rooted human teeth having at least 14 mm of root length, with their coronal aspect decoronated from the cemento-enamel junction. The selected teeth were then randomized into 6 groups (*n* = 15/group) on the basis of different posts (zirconia/glass fiber) and Er,Cr:YSGG laser tips (axial and radial) as shown in Table 1. The bond strength was evaluated using push-out tests for both posts and laser irradiation conditions.

The root canals of all teeth were performed using a crown-down technique from mechanical ProTaper Universal (Dentsply, Bellaigues, Switzerland) rotary files and irrigated with 2 mL of 2.5% sodium hypochlorite. A #35 manual K file (Dentsply) was used as the master file. The root canals were irrigated using distilled water, and then filled with standardized gutta-percha points (Dentsply) and sealed using a root canal sealer with AH Plus (Dentsply, Konstanz, Germany). All treated roots were stored in a humid environment at 37°C for 24 h.

A 10 mm glass fiber post (Postec plus #3; Ivoclar, Schaan, Liechtenstein) was used in the study. With the help of a consecutive sequence of Gates Glidden with increasing diameter, the coronal gutta-percha was removed and the space was enlarged for easier placement. A low-speed bur was used to finish and shape the post space provided in the kit. Subsequently, 2 mL NaOCl was used to irrigate the root canal and later neutralized by 5 mL of distilled water.

An Er,Cr:YSGG laser system (Waterlase; Biolase, San Clemente, CA) with a wavelength of 2780 nm and frequency rate of 20 Hz was used. The two types of laser fiber tips provided radial and axial irradiation. A preset water:air ratio of 37 : 34% for the exchangeable axial fiber tip (400 *μ*m, Biolase) was used. For the radial fiber tip, a 17 mm long sapphire endolase RTF3, 415 *μ*m Biolase was used. The laser parameters were: power, 1.2 W; frequency, 15 Hz; energy fluence, 59.14 J/cm^2^; and pulse duration, 140 *μ*s. The fiber tip was inserted inside the root canal till the apex and moved from the apical to the coronal direction with the average speed of 2 mm/s for 5 times with 20 s interval to supply enough energy to the dentin tissue.

The surfaces of all posts were etched using 37% phosphoric acid for 60 s and later rinsed with water, air-dried, coated with a layer of Monobond primer (Ivoclar, Schaan, Liechtenstein), and then again air-dried for 15 s.

The zirconia and glass fiber posts were then cemented inside their respective post spaces with (test groups) and without laser irradiation (control group), which was carried out manually under pressure for 1-2 min. Light curing was performed on all the specimens for 60 s at a power density of 500 mW/cm^2^ (Optilight plus; Gnatus, Ribeirão Preto, Brazil); the specimens were then stored in containers under maximum humidity at room temperature for 48 h. For cementation, RelyX Unicem Aplicap (3M ESPE AG, Seefeld, Germany) capsules were activated (Aplicap Activator; 3M ESPE AG) and mixed in a high-frequency mixer for 20 s. After the cleaning process, the cement was applied with the elongation tip, and the fiber post was settled.

The specimens were prepared for push-out testing. With the help of a cutting machine (Accutom 5; Struers, Cleveland, OH), six slices (2 on each cervical, middle, and apical) of approximately 1 mm thickness were sectioned for all roots on the plane perpendicular to the long axis of the post. The bonding surface area of the two posts for each slice was computed according to the conical section:

(1) $$\begin{array}{c} A=\pi \left(R+r\right)\sqrt{{\left(R-r\right)}^{2}+\;H^{2}}, \end{array}$$

where *R* = coronal post radius, *r* = apical post radius, and *H* = slice thickness.

All specimens were placed in a universal testing machine within a centralizing plate to ensure load application at the post center. Load was applied from the apical to cervical region, at a defined crosshead speed of 0.5 mm/min until the maximum failure load was obtained. The push-out strength (*σ*), defined in megapascals (MPa), was derived from the following equation:

(2) $$\begin{array}{c} \sigma =\frac{F}{A}, \end{array}$$

where *F* = force load at failure (Newton) and *A* = conical surface area (mm^2^).

Failure modes were determined by visually assessing fractured surfaces and then later confirmed using a stereo microscope (100x). Failure modes were divided and reported as either “adhesive-post” interface, “adhesive-dentin” interface, or “mixed” types of failure.

## 3. Results

**Figure 1:**
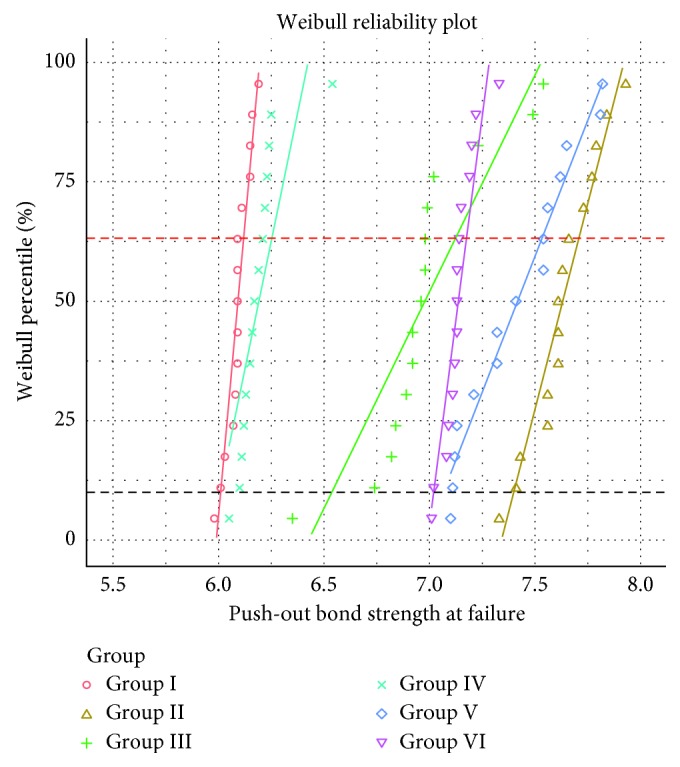
Weibull plots for push-out bond strength and different segments of root dentin.

Normality testing showed all the variables to be normally distributed after the Shapiro–Wilk test. The study groups without laser treatment were designated as I and IV for the glass fiber and zirconia posts, respectively. The axial fiber tips for both glass fiber and zirconia were designated as II and V, whereas III and VI were assigned to glass fiber and zirconia, respectively (Table 1). The highest mean push-out bond strength of glass fiber and zirconia posts was achieved with laser treatment. The highest push-out bond strength was achieved with an axial fiber tip (7.63 ± 1.22 MPa) and the lowest was achieved with a radial fiber tip in the glass fiber group (6.98 ± 0.96 MPa). ANOVA showed a statistically significant difference between the groups (*p*=0.041) (Table 2).  Table 3 presents the comparison of the push-out bond strength values among the groups based on the cervical, middle, and apical segments. The mean push-out bond strength was found to be higher with an axial fiber tip for the cervical and apical segments in both, glass fiber and zirconia groups (*p* < 0.05). The independent *t*-test resulted in the overall highest mean push-out bond strength in the apical segments than in other thirds of the root (*p*=0.026). The middle third showed no statistically significant differences, either between the axial and radial fiber tips or between the glass fiber and zirconia posts (*p*=0.832) (Table 3). Nine failures were seen between the adhesive-dentin interface, while only two failures were seen at the adhesive-post interface. A total of four failures were mixed. Overall, the group with the smallest total number of failure modes was found in the glass fiber post, while the group with the largest number of failure modes was found in the control group (Table 4). The Weibull plot was computed using three Weibull parameters including the Weibull modulus, 10% and 63.2% failure expectation that analyzed differences between the push-out bond strength of the posts among different segments of the root dentin (Table 5). It is noted that the highest value for Weibull modulus was observed in the control group for glass fiber post and radial fiber tip application for the zirconia post, respectively. The Weibull plots of the push-out bond strength and three segments of the root dentin are illustrated in Figure 1. It is observed that the slope is steeper for only radial fiber tip application in the glass fiber posts than the slopes presented for other groups.

## 4. Discussion

This study aimed to evaluate the influence of an Er,Cr:YSGG laser on the bond strength of zirconia and glass fiber posts with root dentin. According to this study, the root system irradiated with Er,Cr:YSGG with an axial fiber tip application achieved the best push-out bond strength in both glass fiber and zirconia dental posts. The possible explanation for the best push-out bond strength could be the complete removal of unnecessary material from the root canal system and a strong bond between the resin cement and the dentin surface. In addition, laser irradiation aids in the removal of the smear layer that helps in the exposure of dentinal tubules and enhancement of mechanical retention, by widely increasing the surface area [23]. Laser irradiation has been widely used for several purposes including disinfection of the dental material surface, improvement in wettability [24], and enhancement of the adhesion and bond strength of the adhesive interfaces [25].

The results of the present study are in accordance with the previous *in vitro* studies [22]. A study that investigated the efficacy of Er,Cr:YSGG laser irradiation on the glass fiber post revealed that the bond strength increased after irradiation. Similarly, this *in vitro* study concluded that laser irradiation at different power settings of the Er,Cr:YSGG laser significantly enhanced the microbond strength of the post core compared with surfaces that did not undergo laser treatment [22].

Research indicates that the efficacy of laser irradiation in increasing the push-out bond strength differs with the use of different laser systems. The surface alteration of a laser-irradiated substrate mainly depends on the laser parameters along with the physical and chemical properties of the material [26]. In our study, the laser parameters were kept constant and the resin cement was employed in the standard manner to lute the zirconia and glass fiber posts. This allowed us to efficiently evaluate and compare the push-out bond strength along with minimizing the probability of bias.

This study also compared the bond strength along different regions of the root canal system. Notably, the bond strength increased in the cervical region of the root canals in the control group compared with the other two regions. This reduction in bond strength in the deeper regions of the root canal system may be attributed to the partial polymerization of the resin cement or an inconsistent adjustment of the dental posts [27]. In the present study, it is also noteworthy that the glass fiber and zirconia posts irradiated with the Er,Cr:YSGG laser revealed an increased bond strength in the apical region. This explains the small diameter of the dental post in the apical region. We hypothesize that an increased removal of the epoxy resin and uncovering of glass fibers and zirconia crystals led to the increased micromechanical retention and penetration of the resin cement, thus augmenting the bond strength.

The present study has some limitations such as the use of a single self-adhesive resin system for luting the dental posts. A comparison of the push-out bond strength after laser irradiation between different adhesive composites would provide the true efficacy of the postadhesive interface. Moreover, the present study did not evaluate the bacterial outcomes after laser therapy. The microbial load after laser irradiation would suggest whether the bacterial count hampers the overall efficacy of laser irradiation in increasing the push-out bond strength. Although a higher bond strength was observed for the zirconia and glass fiber posts, this was not verified with the use of scanning electron microscopy (SEM). The use of SEM would enable us to deeply elucidate the microcracks and shallow pits for micromechanical retention. Therefore, further studies are recommended in this regard. Furthermore, the calculation of bonding surface area may not be standardized and truly applicable on all teeth. This is explained by the various anatomical forms of the root dentin and that root surfaces not being in a perfectly similar cylindrical shape.

## 5. Conclusion

Within the limits of the present study, an enhancement in the push-out bond strength of resin cement, mainly in the cervical region of the root canal, was achieved after irradiation with an Er,Cr:YSGG laser using an axial fiber tip.

## Figures and Tables

**Table 1:** General parameters of the studied groups.

Study groups	Experimental groups		
	Control	Axial fiber tip	Radial fiber tip
Glass fiber posts	I	II	III
Zirconia posts	IV	V	VI

**Table 2:** Overall push-out bond strength values of the different post groups expressed in MPa (±SD).

Study groups	Experimental groups		
	Control	Axial fiber tip	Radial fiber tip
Glass fiber posts	6.09 ± 0.94	7.63 ± 1.22^*∗*^	6.98 ± 0.96
Zirconia posts	6.19 ± 1.00	7.41 ± 1.03^*∗*^	7.13 ± 1.06

^*∗*^Statistically significant difference at *p* < 0.05 compared between the groups.

**Table 3:** Push-out bond strength values of the cervical, middle, and apical segments (MPa) (±SD).

Groups	Cervical segment	Middle segment	Apical segment
Glass fiber posts			
Axial	7.01 ± 1.21^*∗*^	7.14 ± 1.33	7.72 ± 1.99^*∗*†^
Radial	6.82 ± 1.18	7.31 ± 1.09	7.21 ± 1.84

Zirconia posts			
Axial	7.49 ± 1.88^*∗*^	7.01 ± 0.93	7.88 ± 1.92^*∗*†^
Radial	6.52 ± 0.81	7.09 ± 0.09	7.02 ± 1.11

Control group	6.41 ± 2.17	6.14 ± 0.74	5.33 ± 1.00

^*∗*^Statistically significant results obtained by *t*-test at *p* < 0.05 compared between axial and radial fiber tips. ^†^Statistically significant results obtained by *t*-test at *p* < 0.05 compared between each third of the segment.

**Table 4:** Type of failure modes in each group.

Groups	Root segment	Type of failure		
		Adhesive-post	Adhesive-dentin	Mixed
Glass fiber posts	Cervical	0	1	0
	Apical	0	1	0

Zirconia posts	Cervical	1	1	1
	Apical	0	1	0

Control group	Cervical	1	3	2
	Apical	0	2	1

**Table 5:** Mean push-out bond strength of each group together with the results from the Weibull statistical analysis.

Group	*β*	*η*	B10
Group I	127.61	6.12	6.01
Group II	54.68	7.71	7.39
Group III	30.43	7.1	6.59
Group IV	79.41	6.23	6.05
Group V	36.25	7.52	7.07
Group VI	112.52	7.17	7.02

*β*: Weibull modulus; B10 and *η* indicate 10% and 63.2% failure expectation, respectively.

## Data Availability

The statistical data used to support the findings of this study are included within the article.
